# Lymphotoxin-*α* Plays Only a Minor Role in Host Resistance to Respiratory Infection with Virulent Type A *Francisella tularensis* in Mice

**DOI:** 10.1155/2008/239740

**Published:** 2008-08-21

**Authors:** Deng Zhang, Rhonda KuoLee, Greg Harris, Qinxian Zhang, J. Wayne Conlan, Wangxue Chen

**Affiliations:** ^1^National Research Council Canada, Institute for Biological Sciences, 100 Sussex Drive, Room 3100, Ottawa, ON, Canada K1A 0R6; ^2^Basic Medical College of Zhengzhou University, 100 Science Road, Zhengzhou, Henan 450001, China

## Abstract

This study examined the role of lymphotoxin (LT)-*α* in host defense against airborne infection with *Francisella tularensis*, a gram-negative facultative intracellular bacterium and the causative agent of tularemia. Following a low-dose aerosol infection with the highly virulent type A strain of *F. tularensis*, mice deficient in LT*α* (LT*α*−/−) consistently harbored approximately 10-fold fewer bacteria in their spleens at day 2 and 10-fold more bacteria in their lungs at day 4 than LT*α*+/+ mice. However, the mortality and median time to death were indistinguishable between the two mouse strains. In addition, the inflammatory responses to the infection, as reflected by the cytokine levels and leukocyte influx in the bronchoalveolar lavage fluid and histopathological analysis, were generally similar between LT*α*−/− and LT*α*+/+ mice. These data suggest that although LT*α* does not contribute significantly to the resistance and host responses of mice to airborne type A *F. tularensis* infection, it does play a subtle role in the multiplication/dissemination of *F. tularensis*.

## 1. INTRODUCTION

*Francisella tularensis* is a gram-negative facultative intracellular bacterium and the causative agent of tularemia, a systemic infection of many mammals including humans. Left untreated, the virulent type A subspecies of *F. tularensis* routinely caused lethal infection in people particularly after aerosol exposure to the pathogen; as few as 10 virulent type A bacilli can initiate severe disease [1]. Consequently, *F. tularensis* is considered a Category A biological warfare agent. Despite its clinical and biosecurity importance, the molecular basis for the immunopathogenesis of *F. tularensis* infection, particularly when initiated through the respiratory tract, remains largely unknown.

Lymphotoxin-*α* (LT*α*) is a member of the tumor necrosis factor (TNF) superfamily of cytokines and has two distinct roles: as a membrane-bound heterotrimer in combination with LT*β*, it binds the LT*β* receptor and is critical in the development and maintenance of organized secondary lymphoid organs [2], and as a soluble homotrimer, it signals through the TNF receptor pathway and leads to activation of various inflammatory cytokines and chemokines [3, 4]. Indeed, LTs (LT*α* and LT*β*), together with TNF and LIGHT (**L**T-related **i**nducible ligand that competes for **g**lycoprotein D binding to **h**erpesvirus entry mediator on **T** cells), form an integrated signaling network which is important for the regulation of both innate and adaptive immune responses [4]. In this regard, LT*α* has been implicated in the host defense against several different bacterial, viral, and parasitic pathogens (reviewed in [3]). For instance, several studies have shown that after infection with *Mycobacterium tuberculosis*, mice genetically defective in LT*α* (LT*α*−/− mice) harbour increased bacterial burdens and exhibited a shorter median time to death when compared to LT*α*+/+ mice [5–7]. Similarly, mice deficient in LT*α*, LT*β*, and the LT*β* receptor (LT*β*R) are more susceptible to *Listeria monocytogenes* infection than wild-type mice [6, 7]. Given that LT*α* is important in the control of these intracellular bacterial pathogens, in the present study we sought to determine whether it also plays a role in host defense against low-dose aerosol infection with a virulent type A strain of *F. tularensis*.

## 2. MATERIALS AND METHODS

### 2.1. Mice

Eight- to twelve-week old, age-matched B6.129S2-Lt*α* ^tm1Dch^/J (LT*α*−/−), and wild-type C57BL/6J (LT*α*+/+) mice were used in this study. The foundation breeding pairs of LT*α*−/− mice were purchased from Jackson Laboratories (Bar Harbor, Me, USA). Mice were bred and housed under specific-pathogen-free conditions in a federally licensed animal biosafety level-3 facility and given free access to sterile water and certified mouse chow. The animals were maintained and used in accordance with the recommendations of the Canadian Council on Animal Care Guide to the Care and Use of Experimental Animals.

### 2.2. F. tularensis and experimental infections

Stocks of type A *F. tularensis* strain FSC33/snMF, originally isolated from a squirrel in Georgia, USA [8]. For low-dose aerosol exposure, thawed *F. tularensis* stocks were diluted in Mueller Hinton broth containing 20% (v/v) glycerol to maintain infectivity at the high-relative humidity employed. Aerosols of *F. tularensis* strains were generated with a Lovelace nebulizer operating at a pressure of 40 p.s.i. to produce particles in the 4–6 *μ*m range required for inhalation and retention in the alveoli [8]. Mice were exposed to these aerosols for 7 minutes (inhaled dose of ~10 organisms) using a customized commercial nose only exposure apparatus (In-tox Products, Albuquerque, NM) resulting in the implantation of 10–20 organisms into the lungs [8].

### 2.3. Quantitative bacteriology and histopathology

Groups of five mice of each strain were sacrificed at 2 and 4 days after inoculation (dpi) by CO_2_ asphyxiation. Blood samples were collected for the determination of serum cytokine levels. The phenotype of the LT*α*−/− mice was confirmed by visual inspection at necropsy to confirm the absence of peripheral lymph nodes. The lungs and spleens were removed aseptically, cut into small pieces, and then homogenized using an aerosol-proof homogenizer for quantitative bacteriology or fixed immediately by immersion in 10% neutral buffered formalin for histopathology. For quantitative bacteriology, ten-fold serial dilutions of the tissue homogenates were plated on cysteine heart agar supplemented with 1% (w/v) hemoglobin and sulfamethoxazole and trimethoprim. Colonies were counted after 72 hours of incubation at 37°C [8]. For histopathology, the tissues were processed by standard paraffin embedding methods (Department of Pathology and Laboratory Medicine, University of Ottawa, Ottawa, Ontario). Sections were cut 4 *μ*m thick, stained with haematoxylin-eosin (HE) and examined by light microscopy.

### 2.4. Bronchoalveolar lavage (BAL) and cytokine measurements

In some experiments, the lungs were lavaged with five 1.0-ml aliquots of PBS supplemented with 3 mM EDTA [9], and the total lavage cell numbers were counted on a haemocytometer, and differential cell counts were carried out on cytospin preparations stained with Hema3 Stain Set (Fisher Scientific, Middletown, Va, USA). The bronchoalveolar lavage (BAL) fluid was centrifuged at 3000 xg for 7 minutes, supernatants were removed, sterile-filtered, and stored at –80°C. Serum and BAL levels of cytokines and chemokines were determined using Beadlyte Mouse 21-Plex Cytokine Detection System (Upstate, Temecuta, Calif, USA) on a Luminex 100IS system (Luminex, Austin, Tex, USA) [10]. Undiluted BAL and 1 : 2 diluted serum samples (50 *μ*l) were analyzed as specified by the manufacturer (http://www.millipore.com/userguides/tech1/ proto_mpxmcyto-70k). The analysis was done in duplicate, and the cytokine/chemokine concentrations were calculated against the standards using Beadview software (ver 1.03, Upstate).

### 2.5. Statistical analysis

All data are presented as mean ± standard deviation (SD) for each group. Differences between groups were analyzed by two-way ANOVA followed by the Bonferroni multiple comparison test (GraphPad Prism 4.0, GraphPad Software, Inc., San Diego, USA). *P* < .05 was considered to be statistically significant.

## 3. RESULTS AND DISCUSSION

**Figure 1:**
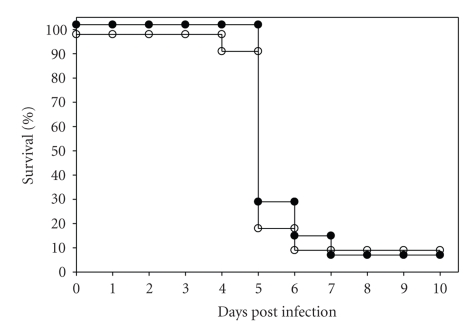
Comparison of the survival rates of LT*α*−/− (open circles) and LT*α*+/+ (closed circles) mice following aerosol inoculation with a low-dose of virulent type A *F. tularensis*. Groups of LT*α*−/− (*n* = 22) and LT*α*+/+ (*n* = 14) mice were challenged by aerosol with type A *F. tularensis* strain FSC033 (inhaled dose of ~10 organisms) and their survival was monitored daily.

Initially, a total of 22 LT*α*−/− and 14 LT*α*+/+ mice were challenged by low-dose aerosol with virulent type A *F. tularensis* and their survival monitored. With the exception of two LT*α*−/− mice and one LT*α*+/+ mouse, all mice succumbed to infection between day 4 and 7 with a median time to death of 5 days (range 4–6 days for LT*α*−/− mice and 5–7 days for LT*α*+/+ mice, *P* > .05 by Kaplan-Meier survival analysis) (Figure 1), indicating that LT*α*−/− mice are no more susceptible to low-dose aerosol challenge with this strain of the pathogen than control LT*α*+/+ mice. It has been previously reported that increased susceptibility of certain immunocompromised mice to intradermal infection with the live vaccine strain (LVS) of *F. tularensis* [11] and oral type A *F. tularensis* infection [10] is only apparent when using a very high inoculum. To examine the possible effect of inoculum size on the need for LT*α* expression to control respiratory infection with type A *F. tularensis*, groups of LT*α*−/− and LT*α*+/+ mice were intranasally challenged with 10, 100, and 1000 cfu type A *F. tularensis* and their survival monitored. This study revealed that the LD_100_ of type A *F. tularensis* for LT*α*−/− and LT*α*+/+ mice was comparable in that all mice died of the infection by dpi 5 (data not shown). These results indicate that LT*α* does not appear to play a significant role in determining the clinical outcome of respiratory infection with various doses of type A *F. tularensis* in mice.

**Figure 2:**
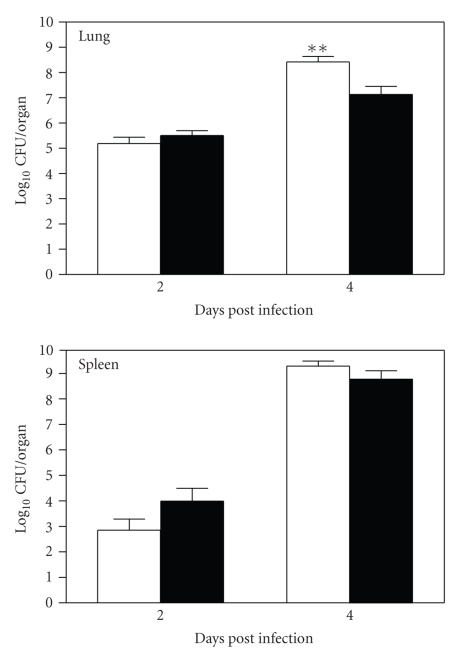
Bacterial burdens in the lungs and spleens of LT*α*−/− (open bars) and LT*α*+/+ (filled bars) mice on days 2 and 4 after aerosol inoculation with a low-dose of type A *F. tularensis* strain, FSC033. The data shown are compiled from two independent experiments with similar results and expressed as mean ± standard deviation (*n* = 8). **P < .01 versus LT*α*+/+ mice.

Since type A strains of *F. tularensis* are extremely virulent for mice even at the minimum challenge dose [8], it remained possible that subtle effects of LT*α* expression were overlooked by the above relatively crude survival experiments. Therefore, we next examined whether LT*α* contributes to the control of *F. tularensis* replication and systemic dissemination by comparing the bacterial burdens in the lungs and spleens of LT*α*−/− and LT*α*+/+ mice at dpi 2 and 4 following aerosol challenge (Figure 2). There was no difference in the bacterial burdens in the lungs, the primary site of infection, between LT*α*−/− and LT*α*+/+ mice at dpi 2. However, the bacterial burdens in the spleens of LT*α*−/− mice were about 1 to 1.5 log, but not statistically significant, lower than those in LT*α*+/+ mice at this time point. By dpi 4, LT*α*−/− mice had approximately 10-fold more bacteria in their lungs than did LT*α*+/+ mice (*P* < .01), and the bacterial burdens in the spleens of LT*α*−/− mice were also higher, although not statistically significant, than those in LT*α*+/+ mice. The subtle differences in bacterial burdens were consistently observed in three independent experiments. Hence, these data imply that although LT*α* is not sufficient to control virulent *F. tularensis* infection, it may play a minor role in the initial dissemination of *F. tularensis* from the lung to spleen and subsequent multiplication of the pathogen in the lungs and spleen.

Histopathologically, both LT*α*−/− and LT*α*+/+ mice showed moderate inflammatory infiltrations in the livers and spleens and mild, focal bronchopneumonia at dpi 2, and by dpi 4 moderately severe necrotic hepatitis, lymphoid follicle destruction in the spleen, and bronchopneumonia. However, as would be expected from the quantitative bacteriology and survival data, no overt differences in tissue histopathology or blood clinical chemistry (data not shown) were observed between LT*α*−/− and LT*α*+/+ mice following aerosol exposure to type A *F. tularensis*.

**Figure 3:**
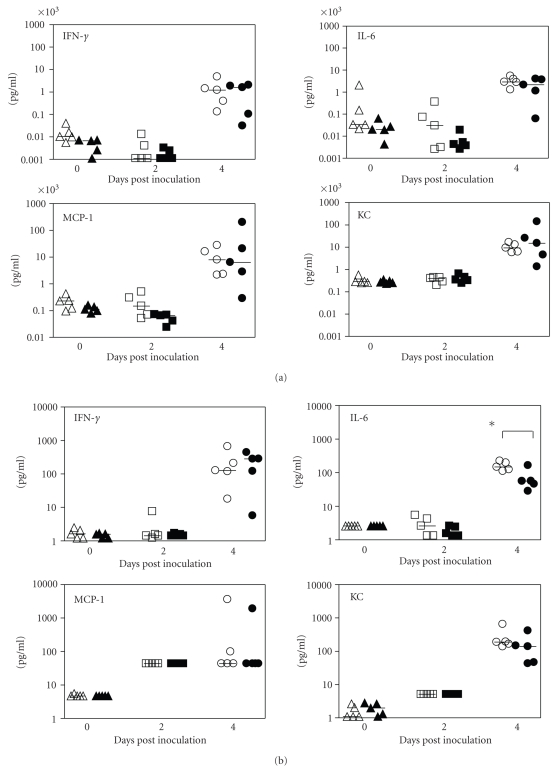
Cytokine and chemokine levels in sera (a) and bronchoalveolar lavage (BAL) fluid (b) of mice inoculated by aerosol with type A *F. tularensis*. Groups of LT*α*−/− (open symbols) and LT*α*+/+ (closed symbols) mice (*n* = 5) were challenged by aerosol with low-dose type A *F. tularensis* strain, FSC033 (inhaled dose of ~10 organisms) on day 0, and blood samples and bronchoalveolar lavage fluid samples were collected at dpi 0, 2, and 4. Cytokine and chemokine levels in the serum and BAL fluid were determined using the Beadlyte Mouse 21-Plex Cytokine Detection System on a Luminex 100 IS instrument. Each symbol represents the corresponding cytokine concentration of an individual mouse. Horizontal lines indicate the median of each group of mice on the indicated post-inoculation days. The detection limits of the assays were <5 pg/ml for both sera and BAL fluid.*P < .05 versus LT*α*+/+ mice.

Early pulmonary recruitment of inflammatory cells and local and systemic production of proinflammatory cytokines are considered important characteristics of innate host responses against respiratory infections including *F. tularensis* [12]. Previous studies have shown that respiratory infection of mice with type A and attenuated live vaccine strain of *F. tularensis* upregulates a number of proinflammatory cytokines, which play important roles in host defense against *F. tularensis* infection [13–15]. Therefore, we determined total and differential leukocyte counts in the BAL fluid to identify the inflammatory cell influx into the lungs on dpi 2 and 4. As can be seen in Table 1, low-dose aerosol infection of mice with type A *F. tularensis* induced no significant difference in either the total cell number or the composition of cell populations (macrophage, neutrophil, and lymphocyte) in the lavage fluids of LT*α*+/+ and LT*α* −/− mice with the exception of a small but not significant increase in lymphocytes in LT*α*−/− mice on both dpi 2 and 4. This is likely due to a higher baseline number of lymphocytes in the lungs of LT*α*−/− mice [16] rather than a result of *F. tularensis* infection. To assess whether LT*α* deficiency alters *F. tularensis*-induced cytokine responses following aerosol challenge with the pathogen, levels of a panel of 21 cytokines and chemokines, including IFN-*γ*, IL-6, KC, and MCP-1, in the BAL and the sera of LT*α*−/− and LT*α*+/+ mice killed at dpi 2 and 4 were measured. Overall, there was little change in the levels of the majority of assayed cytokines in either the BAL or the sera at dpi 2 or 4 in either mouse strain (data not shown). However, *F. tularensis* infection resulted in a substantial increase of MCP-1 and a moderate increase of KC in BAL fluid at dpi 2 (Figure 3(b)) and a substantial increase of IFN-*γ*, IL-6, KC, and MCP-1 in both BAL and sera at dpi 4 (Figures 3(a) and 3(b)), but again no differences were observed between the two mouse strains with the exception of IL-6, which was significantly higher in BAL fluid of LT*α*−/− mice than that of LT*α*+/+ mice (Figure 3(b), *P* < .05).

Recent studies have established that, in addition to its role in the organogenesis of secondary lymphoid organs, LT*α* plays an important role in host defenses against microbial infections (reviewed in [3]). However, the role of LT*α* in host defenses against infection appears to be complex and varies from pathogen to pathogen. In this study, we utilized LT*α*−/− mice and performed some preliminary studies to examine the potential role of LT*α* in the host resistance to respiratory infection with virulent type A *F. tularensis*. Our results showed that LT*α*−/− mice had lower bacterial burdens in their spleens on dpi 2 and higher bacterial burdens in their lungs on dpi 4 when compared to LT*α*+/+ mice but showed no overt differences in clinical outcome, tissue damage, or host immune responses to the infection.

Although our data suggest that LT*α* exerts some subtle influence over the course of aerosol-initiated tularemia, its mechanism of action remains unknown. The possible reasons are as follows: (1) LT-*α* is not crucial in host defense against this pathogen; (2) the role of LT-*α* can be compensated by other cytokines/chemokines in this infection model; and (3) the pathogen is too virulent and even immunocompetent hosts have little resistance to the infection. In this regard, we have previously shown that a number of immunodeficient mice show similar clinical outcome to the immunocompetent mice [17]. The lower bacterial burdens in the spleen of LT*α*−/− mice at dpi 2 could be explained by a delay/reduction in the dissemination of bacteria from the lung since LT*α*−/− mice lack tracheobronchial lymph nodes which normally are the major draining lymph nodes for the lung. Once *F. tularensis* reached the spleens, however, bacteria quickly multiplied and by dpi 4, bacterial burdens were no longer significantly different in the spleens of LT*α*−/− and LT*α*+/+ mice. In fact, LT*α*−/− mice actually seem to have slightly higher burdens in their spleens at this time point despite starting on dpi 2 with substantially lower bacterial burdens (see Figure 2). Also by this time, LT*α*−/− mice harbored significantly more bacteria in their lungs than LT*α*+/+ mice (*P* < .01), suggesting that LT*α* may play a role in host defense against *F. tularensis* infection which is distinct from its role in lymphoid organogenesis. Alternatively, the lack of draining lymph nodes in LT*α*−/− mice may simply cause a delay in antigen presentation leading to a delayed or otherwise impaired antibacterial host response.

In summary, following a low-dose aerosol infection with the highly virulent type A strain of *F. tularensis*, LT*α*−/− mice consistently harbored approximately 10-fold fewer bacteria in their spleens at dpi 2- and 10-fold more bacteria in their lungs at dpi 4 than LT*α*+/+ mice. However, the mortality and median time to death were indistinguishable between the two mouse strains. In addition, the inflammatory responses to the infection, as reflected by the cytokine levels and leukocyte influx in BAL fluid and histopathological analysis, were generally similar between LT*α*−/− and LT*α*+/+ mice. These data suggest that although LT*α* does not contribute significantly to the resistance and host responses of mice to airborne type A *F. tularensis* infection; it does play a subtle role in the multiplication/dissemination of *F. tularensis*.

## Figures and Tables

**Table 1:** Comparison of cell populations in the bronchoalveolar lavage fluid of LT*α*−/− and LT*α*+/+ mice on day 2 and 4 following low-dose aerosol inoculation with type A *F. tularensis*.

Days post-inoculation	Mouse strain	Total cell count (× 10^5^)^(a)^	Differential counts (%)		
			Macrophages	Lymphocytes	Neutrophils
2	LT*α*−/−	2.91 ± 1.59	97.60 ± 0.89	2.00 ± 1.00	0.40 ± 0.55
	LT*α*+/+	1.96 ± 0.31	98.80 ± 0.84	0.40 ± 0.55	0.80 ± 0.84
4	LT*α*−/−	3.02 ± 0.76	95.20 ± 3.27	2.40 ± 1.52	2.40 ± 1.82
	LT*α*+/+	2.70 ± 0.55	94.80 ± 6.76	0.20 ± 0.45	5.00 ± 6.82

^(a)^ The total leukocyte counts are expressed as absolute numbers, and differential counts are expressed as percentages. All data are mean ± standard deviation (*n* = 5) in each group at each time point. No significant differences were observed between LT*α*−/− and LT*α*+/+ mice at any time point.
